# Testing for association between RNA-Seq and high-dimensional data

**DOI:** 10.1186/s12859-016-0961-5

**Published:** 2016-03-08

**Authors:** Armin Rauschenberger, Marianne A. Jonker, Mark A. van de Wiel, Renée X. Menezes

**Affiliations:** Department of Epidemiology and Biostatistics, VU University Medical Center, Amsterdam, 1007 MB The Netherlands; Department of Mathematics, VU University, Amsterdam, 1081 HV The Netherlands

**Keywords:** High-dimensional, Overdispersion, Negative binomial, Global test, Integration, RNA-Seq

## Abstract

**Background:**

Testing for association between RNA-Seq and other genomic data is challenging due to high variability of the former and high dimensionality of the latter.

**Results:**

Using the negative binomial distribution and a random-effects model, we develop an omnibus test that overcomes both difficulties. It may be conceptualised as a test of overall significance in regression analysis, where the response variable is overdispersed and the number of explanatory variables exceeds the sample size.

**Conclusions:**

The proposed test can detect genetic and epigenetic alterations that affect gene expression. It can examine complex regulatory mechanisms of gene expression. The R package *globalSeq* is available from Bioconductor.

**Electronic supplementary material:**

The online version of this article (doi:10.1186/s12859-016-0961-5) contains supplementary material, which is available to authorized users.

## Background

Genetic and epigenetic factors contribute to the regulation of gene expression. A better understanding of these regulatory mechanisms is an important step in the fight against cancer. Of interest are genetic alterations such as single nucleotide polymorphisms (SNPs), copy-number variations (CNVs) and loss of heterozygosity (LOH), as well as epigenetic alterations such as DNA methylation, microRNA expression levels and histone modifications.

From a statistical perspective, it makes sense to represent the expression of one gene as a response variable that changes when some covariates are altered. As a starting point, we assume that all covariates come from a single genetic or epigenetic molecular profile. Typically, more covariates are of interest than there are samples.

A plethora of methods for the analysis of gene expression and covariates has emerged in the last years. Many of these methods test each covariate individually, and subsequently correct for multiple testing or rank the covariates by significance. An alternative approach is the global test from Goeman et al. [1]. The global test does not test the individual but the joint significance of covariates. It allows for high dimensionality, reduces the multiple testing burden, and successfully detects small effects that encompass many covariates. Due to its desirable properties, the global test has become a widely used tool in genomics (e.g. [2–4]).

Currently, gene expression microarrays are being supplanted by high-throughput sequencing. The negative binomial distribution seems to be a sensible choice for modelling RNA sequencing data [5, 6]. One of its parameters describes the dispersion of the variable. If this parameter is unknown, the negative binomial distribution is not in the exponential family. As the global test from Goeman et al. [1] is limited in its current form to the exponential family of distributions, a new test is needed for RNA-Seq data. We will provide here such a test.

After proposing a global test for the negative binomial setting, we perform a simulation study, and analyse two publicly available datasets. The first application concentrates on method validation, overdispersion, and individual contributions. The second application concentrates on robustness against multicollinearity, the method of control variables, and the simultaneous analysis of multiple molecular profiles.

Although we focus on RNA-Seq gene expression data, the test developed here is applicable whenever associations between a count variable and large sets of quantitative or binary variables are of interest. In essence, it can be applied to any other type of sequencing data, such as ChIP-Seq (chromatin immunoprecipitation), microRNA-Seq or meth-Seq (methylation).

## Methods

### The random-effects model

The human genome contains several thousand protein-coding genes. In the following, only one gene is considered at a time. Accordingly, the expression of one gene across all samples is our response variable ***y***=(*y*_1_,…,*y*_*n*_)^*T*^. If we were interested whether a given subset of SNPs affected gene expression, these SNPs would be our *p* covariates. The *n*×*p* covariate matrix ***X*** is potentially high-dimensional (*p*≫*n*).

We represent the relationship between the response and the covariates using the generalised linear model framework from McCullagh and Nelder [7]:

$$\mathrm{E}[y_{i}]=h^{-1}\left(\alpha+\sum\limits_{j=1}^{p} X_{ij} \beta_{j}\right), $$

where *h*^−1^ is an inverse link function, *α* is the unknown intercept, *X*_*ij*_ is the entry in the *i*^*th*^ row and *j*^*th*^ column of ***X***, and *β*_1_,…,*β*_*p*_ are the unknown regression coefficients. This model holds for all samples *i* (*i*=1,…,*n*).

We are interested in testing the joint significance of all regression coefficients. This is challenging because the regression coefficients cannot be estimated by classical regression methods if there are more covariates than samples. Goeman et al. [1] took a novel approach for testing *H*_0_:*β*_1_=…=*β*_*p*_=0 against *H*_1_:*β*_1_≠0∪…∪*β*_*p*_≠0. The decisive step from Goeman et al. [1] was to assume ***β***=(*β*_1_,…,*β*_*p*_)^*T*^ to be random, with the expected value E[***β***]=***0*** and the variance-covariance matrix Var[***β***]=*τ*^2^***I***, where ***I*** is the *p*×*p* identity matrix and *τ*^2^≥0. Then a random-effects model is obtained:

(1) $$ \mathrm{E}\left[y_{i}|r_{i}\right]= h^{-1}(\alpha+r_{i}), \qquad \qquad r_{i} = \sum\limits_{j=1}^{p} X_{ij} \beta_{j}.  $$

This random-effects model allows to rephrase the null and the alternative hypotheses. Defining the random vector ***r***=(*r*_1_,…,*r*_*n*_)^*T*^, it can be deduced that E[***r***]=***0*** and Var[***r***]=*τ*^2^***XX***^*T*^. Now the null hypothesis of no association between the covariate group and the response is given by *H*_0_: *τ*^2^=0. To construct a score test against the one-sided alternative hypothesis *H*_1_: *τ*^2^>0, we need to assume a distribution for *y*_*i*_|*r*_*i*_.

### The testing procedure

We assume the negative binomial distribution *y*_*i*_|*r*_*i*_∼NB(*μ*_*i*_,*ϕ*), where the mean parameter *μ*_*i*_ depends on the sample, but the dispersion parameter *ϕ* does not. We parametrise the negative binomial distribution such that E[*y*_*i*_|*r*_*i*_]=*μ*_*i*_ and $\text {Var}[y_{i} | r_{i}]=\mu _{i}+\phi {\mu _{i}^{2}}$. Its density function is given by

$$f(y_{i})=\frac{\Gamma\left(y_{i}+\frac{1}{\phi}\right)}{\Gamma\left(\frac{1}{\phi}\right) \Gamma(y_{i}+1)} \left(\frac{1}{1+\mu_{i}\phi}\right)^{\frac{1}{\phi}} \left(\frac{\mu_{i}}{\frac{1}{\phi}+\mu_{i}}\right)^{y_{i}}. $$

Various link functions come into consideration for the negative binomial model. We favour the logarithmic link in order to relate the negative binomial model directly to the Poisson model (see below). As library sizes can be unequal, we include the offset $\log (m_{i} / \overline {m})$, where *m*_*i*_ denotes the library sizes, and $\overline {m}$ their *geometric* mean. Thus the mean function becomes

(2) $$  \mu_{i}= \exp \left(\alpha + r_{i} + \log \frac{m_{i}}{\overline{m}}\right)=\frac{m_{i}}{\overline{m}} \exp(\alpha+r_{i}).  $$

When *τ*^2^ is close to zero, the score test is the most powerful test of the null hypothesis *H*_0_:*τ*^2^=0 against the alternative hypothesis *H*_0_:*τ*^2^>0 [8]. Here the score function is the first derivative of the logarithmic marginal likelihood with respect to *τ*^2^. Intuitively, if the marginal likelihood reacts sensitively to changes in *τ*^2^ close to 0, there is evidence against *τ*^2^=0. Using results from le Cessie and van Houwelingen [9], we show in the Additional file 1 how to calculate the score function. This function contains the unknown parameters *α* and *ϕ*, but they can be estimated by maximum likelihood. Replacing the unknown parameters by their estimates leads to the test statistic

(3) $$ \begin{aligned} u_{nb} =& \left\{\sum\limits_{i=1}^{n} \sum\limits_{k=1}^{n} \frac{R_{ik}}{2}~ \frac{(y_{i}- \hat{\mu}_{i}) (y_{k}- \hat{\mu}_{k})}{(1+\hat{\phi}\hat{\mu}_{i})(1+\hat{\phi}\hat{\mu}_{k})} \right\}\\ &- \sum\limits_{i=1}^{n} \frac{R_{ii}}{2}~ \frac{(\hat{\mu}_{i} + y_{i} \hat{\phi} \hat{\mu}_{i})}{(1+\hat{\phi}\hat{\mu}_{i})^{2}}, \end{aligned}  $$

where *R*_*ij*_ is the entry in the *i*^*th*^ row and *j*^*th*^ column of the *n*×*n* matrix ***R***=(1/*p*)***XX***^*T*^, and $\hat {\mu }_{0,i}=(m_{i} / \overline {m}) \exp (\hat {\alpha })$ is the estimated mean under the null hypothesis. For simplicity we always write $\hat {\mu }_{i}$ instead of $\hat {\mu }_{0,i}$. In the Additional file 1 the test statistic is rewritten in matrix notation.

Statistical hypothesis testing depends on the null distribution of the test statistic *u*_*nb*_, which is unknown. We will obtain *p*-values by permuting the response ***y***=(*y*_1_,…,*y*_*n*_)^*T*^ together with the mean $\boldsymbol {\hat {\mu }}=(\hat {\mu }_{1},\ldots,\hat {\mu }_{n})^{T}$. Since this is a one-sided test [10], if the observed test statistic is larger than most of the test statistics obtained by permutation, there is evidence against the null hypothesis.

As we are not using a parametric form for the null distribution of the test statistic, no adjustments for the estimation of *α* and *ϕ* are necessary. Furthermore, maximum likelihood estimation does not depend on the order of the elements in ***y***=(*y*_1_,…,*y*_*n*_)^*T*^. Because neither $\hat {\alpha }$ nor $\hat {\phi }$ vary under permutation, computational efficiency can be achieved with these parameters as given.

When testing for associations between RNA-Seq data and another molecular profile, numerous genes might be of interest. Because one test is performed per gene, the multiple testing problem reappears. (In the applications from below we omit multiple testing correction when analysing the distribution of *p*-values.)

### Relation to the poisson model

For comparison we also consider the Poisson distribution *y*_*i*_|*r*_*i*_∼Pois(*μ*_*i*_) with E[*y*_*i*_|*r*_*i*_]=Var[*y*_*i*_|*r*_*i*_]=*μ*_*i*_ and a logarithmic link function. Proceeding as above we obtain the test statistic

(4) $$ u_{pois} = \left\{ \sum\limits_{i=1}^{n} \sum\limits_{k=1}^{n} \frac{R_{ik}}{2} (y_{i} - \hat{\mu}_{i})(y_{k} - \hat{\mu}_{k}) \right\} - \sum\limits_{i=1}^{n} \frac{R_{ii}}{2} \hat{\mu}_{i},  $$

where the estimates $\hat {\mu }_{i}$ are the same as in the negative binomial model.

In the case of $\hat {\phi }\boldsymbol {\hat {\mu }} = \boldsymbol {0}$ we would have *u*_*nb*_=*u*_*pois*_, but in practice only situations with $\boldsymbol {\hat {\mu }}>\boldsymbol {0}$ are of interest. The fact that $\hat {\phi } = 0$ implies *u*_*nb*_=*u*_*pois*_ is convenient since a negative binomial distribution with a dispersion parameter close to zero is practically equivalent to a Poisson distribution.

### Individual contributions

Following Goeman et al. [1], the test statistic *u*_*nb*_ can be rewritten to reveal the influence of individual samples and covariates.

The contribution of sample *i* (*i*=1,…,*n*) to the test statistic is

(5) $$ \begin{aligned}{}  s_{i} = & \left\{\sum\limits_{k=1}^{n} \frac{R_{ik}}{2}~ \frac{\left(y_{i}- \hat{\mu}_{i}\right) (y_{k}- \hat{\mu}_{k}) }{(1+\hat{\phi}\hat{\mu}_{i})(1+\hat{\phi}\hat{\mu}_{k})} \right\} - \frac{R_{ii}}{2}~ \frac{(\hat{\mu}_{i} + y_{i} \hat{\phi} \hat{\mu}_{i})}{(1+\hat{\phi}\hat{\mu}_{i})^{2}}. \end{aligned}  $$

If *s*_*i*_ is positive, the sample *i* increases the evidence against the null hypothesis. Though, *s*_*i*_ not only depends on the sample *i*, but through ***R***, $\boldsymbol {\hat {\mu }}$ and $\hat {\phi }$ also on the other samples.

Especially useful is the contribution of covariate *j* (*j*=1,…,*p*) to the test statistic:

(6) $$ c_{j} = \frac{1}{2p} \left\{ \sum\limits_{i=1}^{n} X_{ij} \frac{y_{i} -\hat{\mu}_{i}}{1+\hat{\phi} \hat{\mu}_{i}} \right\}^{2} - \sum\limits_{i=1}^{n} \frac{X_{ij}^{2}}{2p}~ \frac{(\hat{\mu}_{i}+y_{i} \hat{\phi} \hat{\mu}_{i})}{(1+\hat{\phi} \hat{\mu}_{i})^{2}}.  $$

Note that multiplying *c*_*j*_ by *p* gives the *u*_*nb*_ that would have been obtained if only the covariate *j* had been tested. Similar to Goeman et al. [1], the test statistic for a group of covariates is the average of the individual test statistics. If *c*_*j*_ is positive, the covariate *j* increases the evidence against the null hypothesis. Conveniently, *c*_*j*_ is independent of all other covariates.

By construction we have $u_{nb}={\sum \nolimits }_{i=1}^{n}s_{i}$ and $u_{nb}={\sum \nolimits }_{j=1}^{p} c_{j}$. Even though a single hypothesis is tested on the covariate group, these decompositions allow to determine which samples and which covariates are the most influential on the test result. If samples or covariates can be put into categories, decomposing the test statistic and grouping samples by category could visualise how each category contributes to the test results. Similarly, if samples or covariates can be ordered according to some genomic or phenomic criteria, patterns might be detected.

### Method of control variables

One drawback of obtaining *p*-values via permutation is the computational burden. Here we will make use of the work from Senchaudhuri et al. [11] in order to estimate *p*-values efficiently.

The proposed test statistic and the test statistic from Goeman el al. [1] have different advantages: whereas the former adequately models overdispersed count data, the latter has a known asymptotic null distribution. Usually we would obtain an unbiased estimate of the *p*-value using $1/k \sum _{i=1}^{k} \boldsymbol {1}[u_{i} \geq u_{0}]$, where ***1*** is the indicator function and *u*_*i*_ represents the proposed test statistic for a permutation (*i*=1,...,*k*) or for the observed data (*i*=0). Following Senchaudhuri et al. [11], we could also obtain an unbiased estimate using $1/k \sum _{i=1}^{k} \boldsymbol {1}[u_{i} \geq u_{0}] - \boldsymbol {1}[q_{i} \geq q_{0}] + p^{*}$, where *q*_*i*_ and *p*^∗^ are the test statistic and asymptotic value, respectively, from Goeman et al. [1]. If the test statistics *u*_*i*_ and *q*_*i*_ have a strong positive correlation, then this alternative estimate is preciser than the usual estimate [11]. (In the applications from below we only use the method of control variables when explicitly stated.)

### Multiple molecular profiles

Not only SNPs but also other molecular mechanisms regulate gene expression. For instance, aberrant DNA methylation levels in promoter regions can activate oncogenes and inactivate tumour suppressor genes. Thus it could be interesting to test for associations between RNA-Seq gene expression data on one hand, and on the other SNP data as well as methylation data.

Let ***X*** represent the *n*×*p* SNP data matrix, and let ***Z*** represent the *n*×*q* methylation data matrix. The model from Eq. 1 allows to test single covariate sets, leading to the test statistic *u*_*nb*_=*u*(***X***) for SNP data, and to the test statistic *u*_*nb*_=*u*(***Z***) for methylation data.

Menezes et al. [12] provided a test for analysing multiple molecular profiles simultaneously, for responses with a distribution in the exponential family. As the negative binomial distribution with an unknown dispersion parameter is not in the exponential family, we have to adapt this test. Following Menezes et al. [12], we include a second covariate set in the random-effects model from Eq. 1:

(7) $$ {} \mathrm{E}[y_{i}|r_{i}]=~ h^{-1}(\alpha+r_{i}), \qquad \qquad r_{i} = \sum\limits_{j=1}^{p} X_{ij} \beta_{j} + \sum\limits_{j=1}^{q} Z_{ij} \gamma_{j}.  $$

Using the ideas and the notation from above: for the random vectors ***β***=(*β*_1_,…,*β*_*p*_)^*T*^ and ***γ***=(*γ*_1_,…,*γ*_*q*_)^*T*^ we assume E[***β***]=E[***γ***]=0, Var[***β***]=*τ*^2^***I***, Var[***γ***]=*υ*^2^***I*** and Cov[***β***,***γ***]=0, where *τ*^2^≥0 and *υ*^2^≥0. Consequently, the random vector ***r***=(*r*_1_,…,*r*_*n*_)^*T*^ has E[***r***]=***0*** and Var[***r***]=*τ*^2^***XX***^*T*^+*υ*^2^***ZZ***^*T*^. The joint test of both covariate sets is described by

$$H_{0}: \tau^{2} = \upsilon^{2} = 0 \quad \text{versus} \quad H_{1}: \tau^{2} \neq 0 \cup \upsilon^{2} \neq 0. $$

Menezes et al. [12] showed that ignoring the correlation between the individual test statistics entails little loss of power, and proposed to use the sum of the individual test statistic as a joint test statistic. As mean and variance of the individual test statistics should be brought onto the same scales [12], our joint test statistic is

(8) $$ u(\boldsymbol{X},\boldsymbol{Z}) = \frac{u(\boldsymbol{X})-\hat{\mathrm{E}}[u(\boldsymbol{X})]}{\sqrt{\widehat{\text{Var}}[u(\boldsymbol{X})]}} + \frac{u(\boldsymbol{Z})-\hat{\mathrm{E}}[u(\boldsymbol{Z})]}{\sqrt{\widehat{\text{Var}}[u(\boldsymbol{Z})]}}.  $$

Permuting as above, we estimate the first two central moments of *u*(***X***) and *u*(***Z***) under the null hypothesis, and calculate a *p*-value for the joint test. Note that this framework can be extended to an arbitrary number of covariate sets. Under *k* covariate sets the joint test statistic is the standardised sum of *k* individual test statistics.

## Results

### Simulation study

We perform a simulation in order to study the power of the proposed test in various circumstances. Instead of randomly generating covariates, we extract a *n*×*p* covariate matrix ***X*** from the HapMap data (see below) at a random position. This maintains the correlation structure between SNPs, and thereby ensures a realistic noise level. Initially we set all coefficients in ***β***=(*β*_1_,…,*β*_*p*_)^*T*^ equal to zero. Then we randomly select a subset of *r* consecutive coefficients, and assign with the probabilities 80 *%* and 20 *%* the values *s* and 2*s* to them, where *s* is the effect size. Using the relation ***μ***=***Xβ***, we calculate the mean vector ***μ***=(*μ*_1_,…,*μ*_*n*_)^*T*^, and simulate the response vector ***y***=(*y*_1_,…,*y*_*n*_)^*T*^ under the distributional assumption *y*_*i*_∼NB(*μ*_*i*_,*ϕ*). This procedure ensures that ***y*** and ***X*** are associated. If we wanted to obtain comparable data under the null hypothesis, we would shuffle the elements in ***μ***. In either case it is of interest how much evidence the proposed test finds for an association between ***y*** and ***X***.

After simulating numerous response vectors independently and identically, we calculate the specificity and sensitivity of the proposed test at various significance levels, and visualise their relation in a ROC curve. All other things being held equal, we either vary the dispersion parameter *ϕ*, the sample size *n*, the effect size *s*, or the number of non-zero coefficients *r*. In the last case we do not select another subset of coefficients, but shorten or lengthen the original subset. It is reassuring that the area under the curve changes in the expected directions (see Figure A in the Additional file 1) and that the type I error rates are maintained (see Table A in the Additional file 1).

A slight modification of this simulation study allows to compare the statistical power between testing all covariates at once and testing them one by one. For this we extract various covariate matrices ***X*** from the HapMap data, and let the coefficient vector ***β*** exclusively take non-zero values. For each covariate matrix ***X*** we simulate one response vector ***y*** under the alternative hypothesis. Using the proposed test, we test the joint as well as the individual significance of the *p* covariates. Subsequently, we compare the joint *p*-value with the minimum of the FDR-corrected individual *p*-values (false discovery rate correction). In our setting with many small effects, joint testing is more powerful than individual testing (see Table B in the Additional file 1). Note that this might not hold in situations with fewer or stronger effects.

### Application: HapMap

Here we verify that the proposed test finds biologically meaningful signals, examine whether overdispersion is present, and measure the influence of covariates and samples.

We use the datasets from Montgomery et al. [13] and Pickrell et al. [14] that were made available in a preprocessed form by Frazee et al. [15]. They include RNA-Seq gene expression data for 59 individuals from the population “Utah residents with ancestry from northern and western Europe” (CEU) and 69 individuals from the population “Yoruba in Ibadan, Nigeria” (YRI). Excluding genes outside the 22 autosomes, without any variation within the sample, or without annotations, 11 700 genes are left. For each individual, SNP data is obtained from the International HapMap Consortium [16]. Throughout this application we use the term SNP to designate the number of minor alleles per locus (0, 1 or 2), considered quantitatively.

### Stratified permutation test

Considering one gene at a time, its expression level over all individuals is used as a response vector, and the SNPs in the neighbouring region are used as a covariate matrix. The aim is to detect regions where causal SNPs might be. To be precise, we test each of the 11 700 gene expression vectors for associations with the respective SNPs that are within a window of ± 1 000 base pairs around the gene. This window size leads to *p*>*n* for approximately 13 *%* of the genes, with a maximum of *p*=5 152. Under the null hypothesis of no association between gene expression and local SNPs, the *p*-values would follow a uniform distribution.

Each sample either belongs to the population CEU or to the population YRI, and we account for this grouping by restricting permutations to keeping samples within the same population. As the distribution of *p*-values is weakly positively skewed, the overall evidence against the null hypotheses is small (see Figure B in the Additional file 1). Only 40 genes reach the minimal *p*-value given by the reciprocal of the number of permutations (see Table C in the Additional file 1). As in Hulse and Cai [17], we find some genes in the major histocompatibility complex family to be associated with nearby SNPs. Our results display good overlap with the examined results from Lappalainen et al. [18] (see Figure C in the Additional file 1), leading us to conclude that the proposed test identifies biologically meaningful signals.

### Presence of overdispersion

The reliability of the global test depends on how well the underlying distribution of RNA-Seq gene expression data is approximated. We are interested whether this dataset requires a model with an offset as well as an dispersion parameter, or whether a simpler model would be sufficient.

Fitting under the null hypothesis of no association between gene expression and local SNPs, we observe that the Poisson distribution without an offset has a poor fit, and that including an offset for different library sizes or using the negative binomial distribution improves the fit (see Figure D in the Additional file 1).

In this example the Poisson model with an offset seems to fit almost equally well to the data as the negative binomial with or without an offset. This might be caused by genetic homogeneity within populations or by the absence of diseases. In cancer datasets we expect a much higher variability between individuals (see below).

### Individual contributions

For each of the 11 700 tests (one test per gene), the test statistic can be decomposed to show the contribution of individual samples or covariates (Eqs. 5 and 6). By construction these contributions can be positive or negative, but the same holds for their expected values under the null hypothesis. We select two tests (i.e. genes) in order to illustrate these decompositions.

**Fig. 1:**
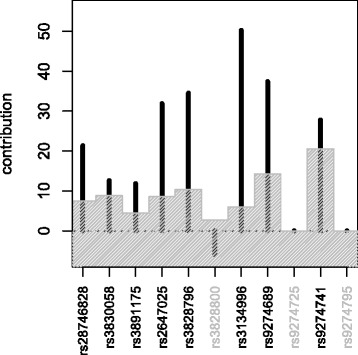
Contributions of covariates to the test statistic for gene *HLA-DQA2*. The *shaded* area indicates their lower 99 *%* confidence interval under the null hypothesis

For gene *HLA-DQA2*, most covariates have a larger influence than expected under the null hypothesis (Fig. 1). This suggests that several SNPs might be associated with the expression of the gene. Indeed, if they are tested individually using 10 000 permutations, almost half of them obtain the minimal *p*-value of 0.0001.

**Fig. 2:**
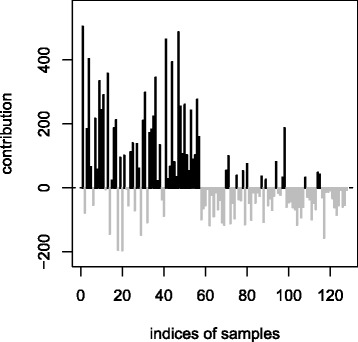
Contributions of samples to the test statistic for gene *CIRBP*. Samples 1 to 59 are from population CEU, samples 60 to 128 are from population YRI

For gene *CIRBP*, the samples from the population CEU tend to contribute positively to the test statistic, whereas those from the population YRI tend to have negative contributions (Fig. 2). Accordingly, the ordinary permutation test would give a much smaller *p*-value than the stratified permutation test (0.001 versus 0.065). In the case of gene *CIRBP* we cannot detect any sample with an extreme contribution to the test statistic.

### Application: TCGA

In this application we illustrate that the proposed test is robust against multicollinearity of the covariates, apply the method of control variables, and test for association with multiple covariate sets simultaneously.

We use a dataset on prostate cancer from TCGA et al. [19]. It includes expression levels of 17 678 genes, DNA methylation levels at 482 486 sites, and DNA copy numbers measured at 30 000 locations for 162 individuals. Section B in the Additional file 1 gives further information about this dataset, including preprocessing. Examining some randomly selected genes, it becomes clear that the Poisson distribution fits badly, but the negative binomial distribution with a free dispersion parameter fits well to the gene expression data (see Figure E in the Additional file 1). Given that the RNA-Seq data has been adjusted for different library sizes, we do not use an offset.

### Robustness to multicollinearity

McCarthy, Chen and Smyth [20] developed a test of differential expression between conditions defined by one or more covariates. Taking the design matrix into account when estimating the dispersion parameters, this generalised linear model likelihood-ratio test is powerful for testing small numbers of covariates jointly. However, as in all regression models, multicollinearity may have undesirable consequences.

When testing for associations between gene expression and local genetic or epigenetic variations, high-dimensional situations can occur. Then the likelihood-ratio test breaks down due to singularity, but the global test is still applicable.

But also in low-dimensional situations perfect multicollinearity poses a practical problem. For example, copy number data has a relatively high chance of being perfectly multicollinear, because it correlates highly between locations. If we wanted to apply the likelihood-ratio test nonetheless, we would have to drop some covariates. In contrast, the global test exploits this correlation.

### Method of control variables

Here we compare the method of control variables with the crude permutation test, based upon randomly selected genes. Testing the expression of each gene for associations with copy numbers that are within 1 000 000 base pairs around the gene, we estimate the precision of the estimated *p*-values by repeating each permutation test many times. The precision of the estimated *p*-values not only increases with the number of permutations, but according to Table 1 also when switching from the crude permutation test to the method of control variables. For the genes (i.e. tests) in Table 1 the correlation between the two test statistics is sufficiently strong to make this happen, but this is not necessarily true for all genes. However, also in the application HapMap this improvement occurs at all randomly selected genes (see Table D in the Additional file 1). Before deciding between the two methods, we advise to estimate the precision analytically [11].

**Table 1:** Precision of estimated *p*-values from tests with 100 permutations, estimated from 1,000 repetitions

	EXOSC9	FRMD1	SLC22A6	CNFN	PDHB	U2AF1L4
crude	3.50E+03	4.25E+02	4.13E+02	5.74E+03	1.75E+03	3.38E+03
MCV	2.78E+04	9.17E+04	1.29E+03	1.28E+13	1.89E+04	1.43E+04
	ENTPD6	TMED2	POU6F1	ANP32E	CLDND1	C2orf54
crude	1.91E+03	1.61E+03	1.67E+03	1.38E+05	3.91E+03	5.57E+02
MCV	7.31E+03	8.47E+03	1.50E+04	7.24E+10	3.93E+04	1.12E+03

At all randomly selected genes (*columns*) the crude permutation test (*first row*) is outperformed by the method of control variables (*second row*) in terms of precision

### Multiple molecular profiles

Several molecular mechanisms are believed to have an impact on gene expression. In the following, the simultaneous analysis from Eqs. 7 and 8 is applied to chromosome 1. We test for associations between RNA-Seq gene expression data on one hand, and on the other methylation values within ± 50 000 base pairs, or copy numbers within ± 2 000 000 base pairs around the start location of the gene. To make the comparison meaningful, the same 1 000 permutations are used for the individual tests and the joint test.

**Fig. 3:**
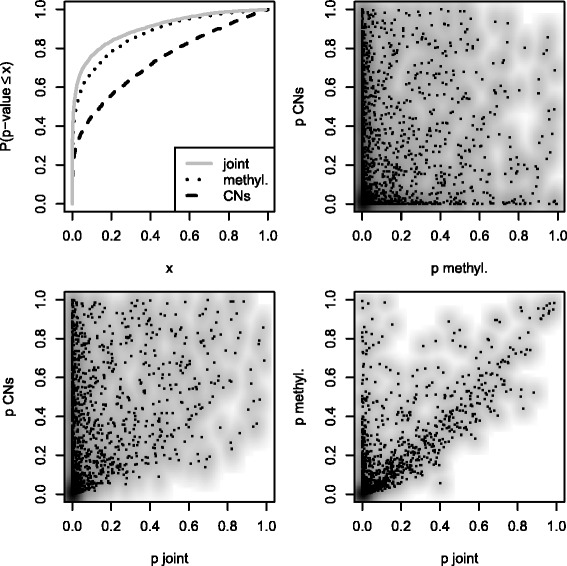
Empirical cumulative distribution functions and scatterplots of *p*-values. We test for associations between RNA-Seq on one hand, and either copy numbers, methylations or both on the other. The corresponding Spearman correlation coefficients are 0.04 (*top right*), 0.55 (*bottom left*) and 0.72 (*bottom right*)

Figure 3 shows: (1) the evidence against null hypotheses is stronger for methylations than for copy numbers; (2) testing methylations and copy numbers jointly leads to an increase in power compared to testing only copy numbers or only methylations; (3) the joint *p*-values are strongly correlated with both sets of individual *p*-values.

**Fig. 4:**
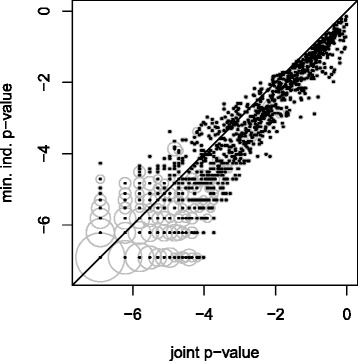
Scatterplot of logarithmic *p*-values from the simultaneous analysis of multiple covariate sets. *Black points* match the minimal individual *p*-values with the corresponding joint *p*-values. *Grey circles* visualise how often these combinations occur

Because window sizes are arbitrary, great care is required for biological interpretations of (1). However, (2) and (3) imply that the joint test adds some information to the individual tests. Indeed, in 13 *%* of the cases the joint test gives smaller *p*-values than both individual tests (Fig. 4). This illustrates the fact that the joint test finds effects that are missed by *both* individual tests. At a false discovery rate of 5 *%*, Table E in the Additional file 1 lists all genes that are insignificant in both individual tests but significant in the joint test. Extreme examples are the genes *CNKSR1*, *ZNHIT6*, *TMEM56*, *PRPF38B*, and *SLC39A1*, where both individual *p*-values are larger than 0.005, but the joint *p*-values are equal to 0.001. Among these genes, *ZNHIT6* and *SLC39A1* have been linked to prostate or breast cancer [21].

## Discussion

We have proposed a test for association between RNA-Seq data and other molecular profiles. By virtue of the negative binomial distribution, we have accounted for overdispersion in the RNA-Seq data. And owing to a random-effects model, we have allowed for the high dimensionality of the other molecular profiles. Varying library sizes are naturally dealt with by an offset in the model.

We applied the proposed test to detect regulatory mechanisms of gene expression. Thereby we illustrated some of its advantages: (1) stratified permutation allows to account for simple groupings; (2) if overdispersion is absent, the proposed test is equivalent to the one based on the Poisson distribution; (3) the test statistic can be decomposed to show the influence of covariates or samples; (4) the test is applicable in presence of multicollinearity; (5) an extension allows to analyse multiple covariate sets simultaneously.

We use simple offsets and dispersion estimates, but more sophisticated results can easily be integrated into the proposed test. In this regard, sharing information on overdispersion would probably improve the performance of the test under small sample sizes.

The proposed test is based on permutations. Due to the lower multiple testing burden, testing the joint significance of covariates requires much less permutations than testing their individual significance. Even though the computation time for a single test is usually much shorter than one second, genome-wide analyses can be computationally expensive. Running several processes in parallel and interrupting permutation when it becomes impossible to reach a predefined significance level [22] reduces the computation time of a genome-wide analysis to a couple of minutes. If expressions for the mean and the variance of the test statistic were obtained, it would be possible to approximate its null distribution without using permutations. This would allow to obtain significant *p*-values under small sample sizes, and lead to a drastic reduction of computation time. An alternative way of achieving precision as well as speed is the discussed method of control variables.

## Conclusions

We have proposed a powerful test for finding eQTL effects based upon RNA-Seq data. It can be computed efficiently and is able to handle sets of highly correlated covariates.

## Software

The R package *globalSeq* runs on any operating system equipped with R-3.3.0 or later. It is available from Bioconductor under a free software license: http://bioconductor.org/packages/globalSeq/.

## Additional file

Additional file 1
**Appendix.** Mathematical details, supplementary plots and information on reproducibility. (PDF 487 kb)
