# Supplementary material for: Testing for association between RNA-Seq and high-dimensional data
Source: BMC Bioinformatics. 2016 Mar 8;17:118. doi: 10.1186/s12859-016-0961-5 (PMC4782413; doi:10.1186/s12859-016-0961-5)
Supplement: Additional file 1 — Appendix. Mathematical details, supplementary plots and information on reproducibility. (PDF 487 kb) [file 12859_2016_961_MOESM1_ESM.pdf]

Testing for association between RNA-Seq and high-dimensional data - Appendix

A Rauschenberger, **MA Jonker**, **MA van de Wiel** and **RX Menezes**

A Derivation of the test statistic

Here we derive the proposed test statistic. Note that a more intuitive explanation of the setting is given in the paper.

Setting

The setting is similar to Goeman et al. (2004). Denote the response across all samples by $\boldsymbol{y} = (y_1, \ldots, y_n)^T$, and the library sizes by $\boldsymbol{m} = (m_1, \ldots, m_n)^T$. Each y_i is modelled by $\mathrm{E}[y_i|r_i] = \gamma_i \exp(\alpha + r_i)$, where α is the intercept, $\log(\gamma_i)$ an offset, and r_i a realisation of the random effect. We use $\gamma_i = m_i/\overline{m}$, where $\overline{m} = (\prod_{i=1}^{n} m_i)^{(1/n)}$. For the random vector $\boldsymbol{r} = (r_1, \ldots, r_n)^T$ we assume $\mathrm{E}[\boldsymbol{r}] = 0$ and $\mathrm{Var}[\boldsymbol{r}] = \tau^2 \boldsymbol{X}\boldsymbol{X}^T$, where $\boldsymbol{X}$ is the $n \times p$ covariate matrix. The aim is to test $H_0 : \tau^2 = 0$ against $H_1 : \tau^2 > 0$. For simplicity we define $\boldsymbol{R} = (1/p)\boldsymbol{X}\boldsymbol{X}^T$ and let R_{ij} denote the element in the ith row and j^{th} column of $\boldsymbol{R}$.

Distribution

We assume $y_i|r_i \sim \mathrm{NB}(\mu_i, \phi)$, where $\mu_i > 0$ and $\phi > 0$ for all $i = 1, \ldots, n$. Under the chosen parametrization $\mathrm{E}[y_i|r_i] = \mu_i$ and $\mathrm{Var}[y_i|r_i] = \mu_i + \phi\mu_i^2$. The density function is:

$$f_i(y_i|\mu_i, \phi) = \frac{\Gamma\left(y_i + \frac{1}{\phi}\right)}{\Gamma\left(\frac{1}{\phi}\right)\Gamma(y_i + 1)} \left(\frac{1}{1 + \mu_i\phi}\right)^{\frac{1}{\phi}} \left(\frac{\mu_i}{\frac{1}{\phi} + \mu_i}\right)^{y_i},$$

$$\text{where} \quad \log(\mu_i) = \log\{\mathrm{E}[y_i|r_i]\} = \alpha + r_i + \log(\gamma_i),$$
$$\mu_i = \mathrm{E}[y_i|r_i] = \gamma_i e^{\alpha + r_i}.$$

Score

Le Cessie and van Houwelingen (1995) show how to obtain the score for testing $H_0 : \tau^2 = 0$ against $H_1 : \tau^2 > 0$. The calculations from le Cessie and van Houwelingen (1995) start with the marginal likelihood function:

$$L(\alpha, \tau^2) = \mathrm{E}_r \left[\prod_{i=1}^{n} f_i(y_i | r_i, \alpha, \tau^2) \right].$$

The crucial step of le Cessie and van Houwelingen (1995) is to take the Taylor expansion with respect to the random effect before taking the expectation. Differentiating this approximation of $L(\alpha, \tau^2)$ with respect to τ^2, and evaluating the result at $\tau^2 = 0$ gives the score. Under the null hypothesis only some terms of the score can be different from zero:

$$u^*_{nb} = \left\{ \sum_{i=1}^{n} l_i^{(2)}(0) \frac{R_{ii}}{2} \right\} + \sum_{i=1}^{n} l_i^{(1)}(0) \sum_{j=1}^{n} l_j^{(1)}(0) \frac{R_{ij}}{2},$$

where
$$l_i^{(1)}(r_i) = \frac{\partial \log \{ f_i(y_i | r_i, \alpha, \phi) \}}{\partial r_i} = \frac{y_i - \gamma_i e^{\alpha + r_i}}{1 + \phi \gamma_i e^{\alpha + r_i}},$$
$$l_i^{(2)}(r_i) = \frac{\partial^2 \log \{ f_i(y_i | r_i, \alpha, \phi) \}}{\partial r_i^2} = \frac{-\gamma_i e^{\alpha + r_i} - y_i \phi \gamma_i e^{\alpha + r_i}}{(1 + \phi \gamma_i e^{\alpha + r_i})^2}.$$

Plugging the expressions for $l_i^{(1)}(0)$ and $l_i^{(2)}(0)$ into u^*_{nb} leads to

$$u^*_{nb} = \left\{ \sum_{i=1}^{n} \frac{-\gamma_i e^{\alpha} - y_i \phi \gamma_i e^{\alpha}}{(1 + \phi \gamma_i e^{\alpha})^2} \frac{R_{ii}}{2} \right\} + \sum_{i=1}^{n} \frac{y_i - \gamma_i e^{\alpha}}{1 + \phi \gamma_i e^{\alpha}} \sum_{k=1}^{n} \frac{y_k - \gamma_k e^{\alpha}}{1 + \phi \gamma_k e^{\alpha}} \frac{R_{ik}}{2}.$$

Parameter estimation

Under the null hypothesis we have $y_i \sim NB(\mu_i, \phi)$ where $\mu_i = \gamma_i \exp(\alpha)$. Maximum likelihood estimation leads to $\hat{\alpha} = \log(\bar{y}) - \log(\bar{\gamma})$. The maximum likelihood estimate for the dispersion parameter ϕ can be obtained by numeric maximisation.

Test statistic

In matrix notation the test statistic is

$$u_{nb} = \frac{1}{2}(\boldsymbol{y} - \hat{\boldsymbol{\mu}}_{\boldsymbol{0}})^T \boldsymbol{TRT}(\boldsymbol{y} - \hat{\boldsymbol{\mu}}_{\boldsymbol{0}}) - \frac{1}{2}(\hat{\boldsymbol{\mu}}_{\boldsymbol{0}} + \phi \boldsymbol{y} \circ \hat{\boldsymbol{\mu}}_{\boldsymbol{0}}) \boldsymbol{T}^2 \boldsymbol{d},$$

where

$\hat{\boldsymbol{\mu}}_{\boldsymbol{0}}$ is the column vector $(\exp(\hat{\alpha})/\overline{m})\boldsymbol{m}$,

$\boldsymbol{T}$ is the diagonal matrix with the diagonal elements $T_{ii} = 1/(1 + \phi \hat{\mu}_i)$,

$\boldsymbol{y} \circ \hat{\boldsymbol{\mu}}_{\boldsymbol{0}}$ is the entrywise product of the vectors $\boldsymbol{y}$ and $\hat{\boldsymbol{\mu}}_{\boldsymbol{0}}$, and

$\boldsymbol{d}$ is the column vector of the main diagonal of $\boldsymbol{R}$.

B Cancer dataset

Variables

The prostate cancer dataset from TCGA et al. (2013) includes data of various types and on three different levels. We used preprocessed forms of the RNA-Seq data (gene, level 3), of the DNA methylation data (human methylation 450 array, level 3), and of the DNA copy number data (CNV data extracted from SNP array, level 3). The last-mentioned data involves copy numbers measured at equally spaced loci on the genome, obtained from the segmented copy number profiles.

Samples

Our criterion for sample selection was the availability of gene expression, methylation, copy number and single nucleotide polymorphism data. This lead to a sample size of 162 individuals.

Normalisation

TCGA et al. (2013) use MapSplice (Wang et al., 2010) and RSEM (Li & Dewey, 2011) for calculating RNA-Seq gene expression data. The methylation data from TCGA et al. (2013) consists of the calculated beta values, i.e. the ratios between the methylated and the total probe intensities, mapped to the genome. We use the logit transformation to obtain values on the real line. In contrast, we do not modify the normalised copy number data from TCGA et al. (2013).

Batch effects

According to the TCGA batch effects tool (MD Anderson Cancer Center, 2016), the between batch dispersion (DB) is much smaller than the within batch dispersion (DW) in the RNA-Seq gene expression data, the copy number data, and the methylation data. Due to the small dispersion separability criteria (DSC = DB/DW) we do not correct the data for batch effects.

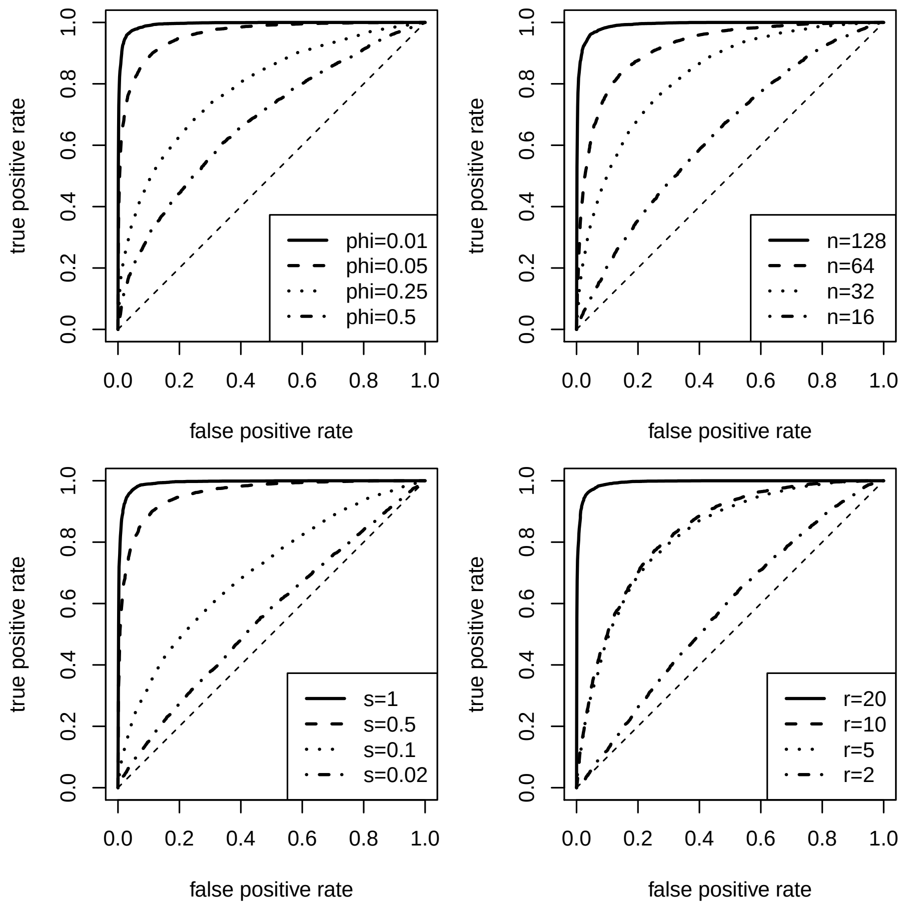

Figure A: **ROC curves from the simulation study.** Given an $128 \times 1\,000$ covariate matrix and a coefficient vector of length $1\,000$, we show how the area under the curve depends on the dispersion parameter (default $\phi = 0.01$), the sample size (default $n = 128$), the effect size (default $s = 1$), and the number of non-zero coefficients (default $r = 20$). At all times only one of the parameters differs from its default value. For each line we simulate $10\,000$ expression vectors, and each expression vector is simulated under the alternative hypothesis with a probability of 50%.

	solid	dashed	dotted	dash-dot
ϕ	0.045	0.052	0.047	0.049
n	0.054	0.053	0.052	0.051
s	0.050	0.051	0.049	0.051
r	0.051	0.051	0.056	0.047

	solid	dashed	dotted	dash-dot
ϕ	0.009	0.008	0.007	0.010
n	0.011	0.011	0.009	0.007
s	0.011	0.009	0.010	0.010
r	0.011	0.010	0.010	0.009

Table A: **Type I error rates in the simulation study.** Under each simulation setup from Figure A we calculate the type I error rates at the 5% (top) and 1% (bottom) significance levels. The row and column names match the entries with the lines in Figure A. As the average rates are 5.1% and 1.0% respectively, there is little concern about rejecting more true null hypotheses than expected. **Additional Note:** In order to verify that the type I error rate is not only maintained across genes, but also for individual genes, we simulate 5000 expression vectors $\boldsymbol{y} = (y_1, \ldots, y_{128})^T$ from the negative binomial distribution with $\mu = 7$ and $\phi = 0.1$. Testing for associations with the given covariate matrix $\boldsymbol{X}$ leads to the type I error rates 4.4% and 0.7% at the 5% and 1% significance levels, respectively.

	$\alpha = 0.01$	$\alpha = 0.05$	$\alpha = 0.10$	$\alpha = 0.20$
joint	0.51	0.67	0.77	0.86
individual	0.31	0.62	0.70	0.79

Table B: **Statistical power of joint and individual testing at various significance levels $\boldsymbol{\alpha}$.** We simulate 1 000 response vectors under the alternative hypothesis ($n = 128$, $p = r = 50$, $s = 1$, $\phi = 0.01$). After testing the covariates jointly as well as individually, we compare the joint p-value with the minimum of the FDR-corrected individual p-values. Joint testing rejects a higher percentage of false null hypotheses than individual testing.

CCDC27	FAM157A	LDHC	CLEC4E	ZNF774	RNF125
BMP8A	HLA-DRB1	APIP	ST8SIA1	RPS2	CLIP3
KTI12	HLA-DQA2	SLC35C1	MTERF2	CDIP1	SPINT4
ID2	HLA-DQB2	ACP2	RAB35	TEKT5	KRTAP8-1
POMC	B4GALT1	PTPMT1	MPHOSPH9	LCAT	SHISA8
KCNK3	NUDT2	YPEL4	GOLGA5	ZSWIM7	
CCR4	FAM24A	LTBR	EMC7	NUFIP2	

Table C: **List of gene symbols.** In the application HapMap these genes obtain the minimal p-value of 0.001 (given by the reciprocal of the number of permutations).

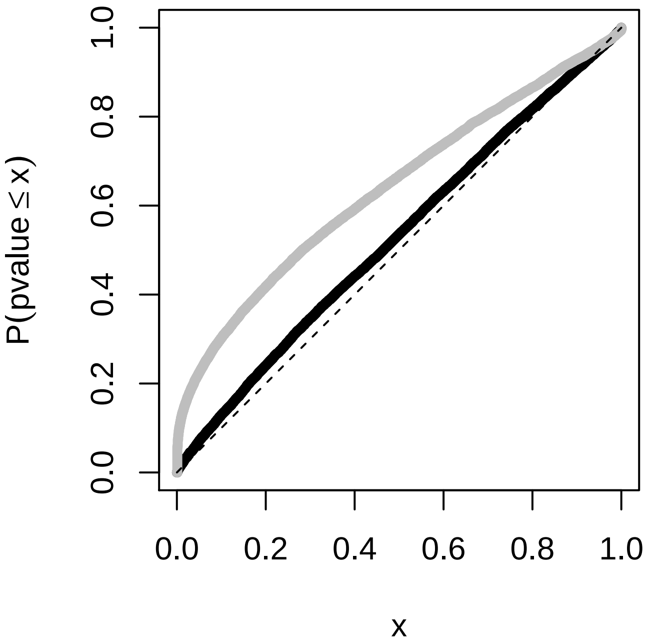

Figure B: **Empirical cumulative distribution plots of p-values from the application HapMap.** At any reasonable significance level, the non-stratified permutation test (grey) rejects more null hypotheses than the stratified permutation test (black). Naturally, genetic variation is high between and low within populations. Ignoring population structure increases genetic variation and thereby statistical power, whereas accounting for population structure decreases bias.

	IL22RA2	C10orf67	HCAR1	ZNF428	V5	RASGRP4
crude	6.03E+02	1.97E+03	4.08E+02	5.07E+02	4.55E+02	8.92E+02
MCV	9.85E+02	4.88E+03	1.55E+03	4.99E+03	7.85E+05	2.71E+03

	DEFB125	KBTBD13	KCNK13	VANGL2	CDKN2AIPNL	CXCL1
crude	8.59E+02	4.06E+02	5.38E+02	4.74E+02	2.03E+03	1.39E+03
MCV	1.43E+04	1.05E+03	3.63E+03	1.77E+03	1.99E+04	7.15E+03

Table D: **Precision of estimated p-values from tests with 100 permutations, estimated from 1 000 repetitions.** At all randomly selected genes from the application HapMap (columns) the crude permutation test (first row) is outperformed by the method of control variables (second row).

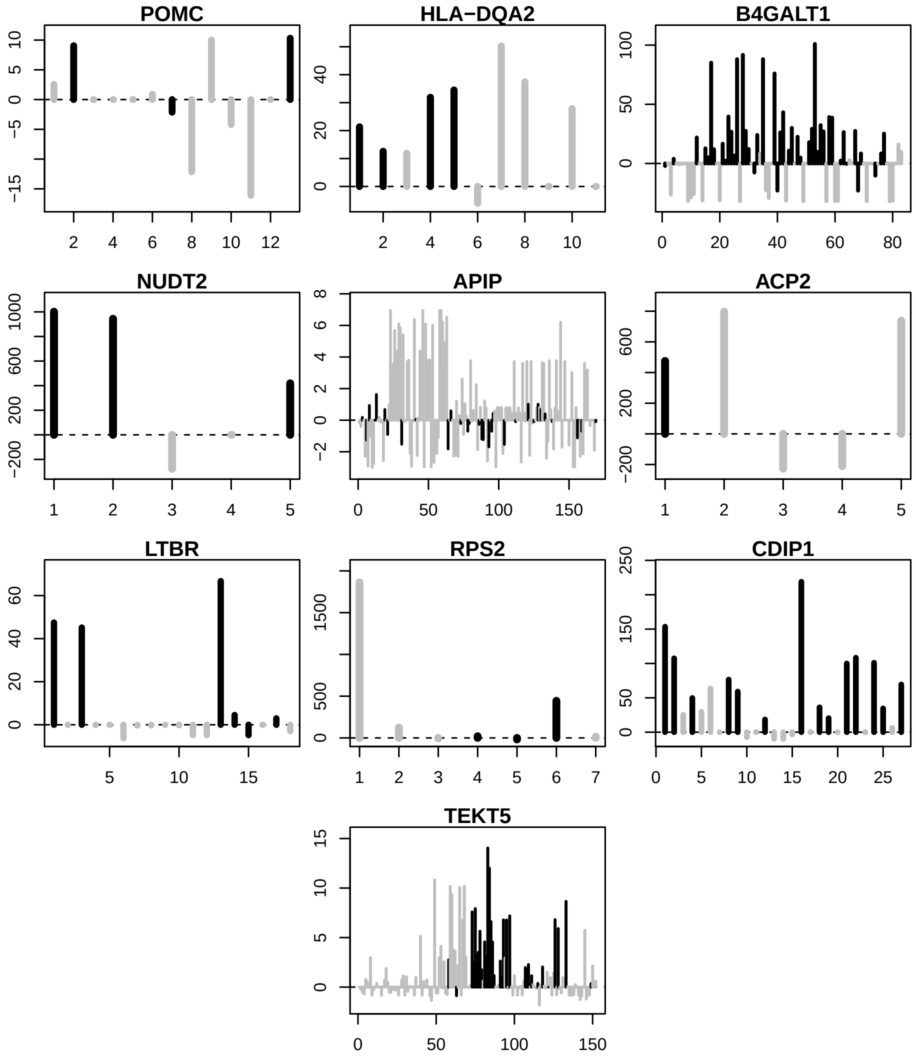

Figure C: **Comparison with known associations.** In the application HapMap only these 10 genes have a covariate group that is declared jointly significant by the proposed test[1] *and* that includes at least one individually significant SNP as found by Lappalainen et al. (2013)[2]. We decompose the corresponding proposed test statistics to obtain the contributions (y-axes) of the individual SNPs (indices on x-axes). Whereas 79% of the individually significant SNPs (black) from Lappalainen et al. (2013) have a positive contribution to the proposed test statistics, this is only true for 45% of the other SNPs (grey).

[1]We obtain a p-value equal to the reciprocal of the number of permutations in the application HapMap. [2]Lappalainen et al. (2013) obtain a p-value below the false discovery rate of 5% in the gene expression analysis of 373 samples from European populations.

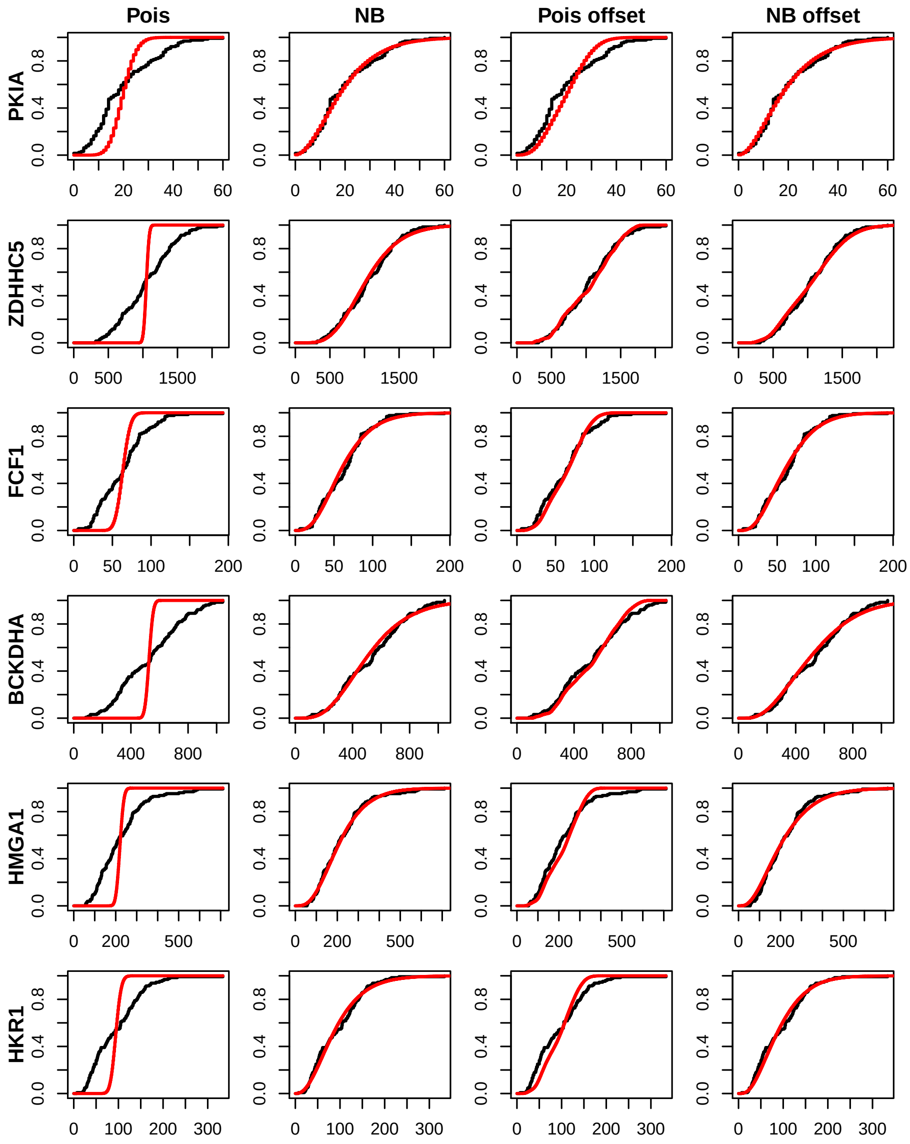

Figure D: **Cumulative distribution functions of RNA-Seq from the application HapMap for randomly selected genes.** Each row represents one gene, and each column represents one model. It is of interest how close the fitted distributions (red) come to the empirical distributions (black). If library sizes are ignored (columns 1 and 2), the negative binomial distribution with a free dispersion parameter has a much better fit than the Poisson distribution. If an offset is included (columns 3 and 4), the differences become smaller.

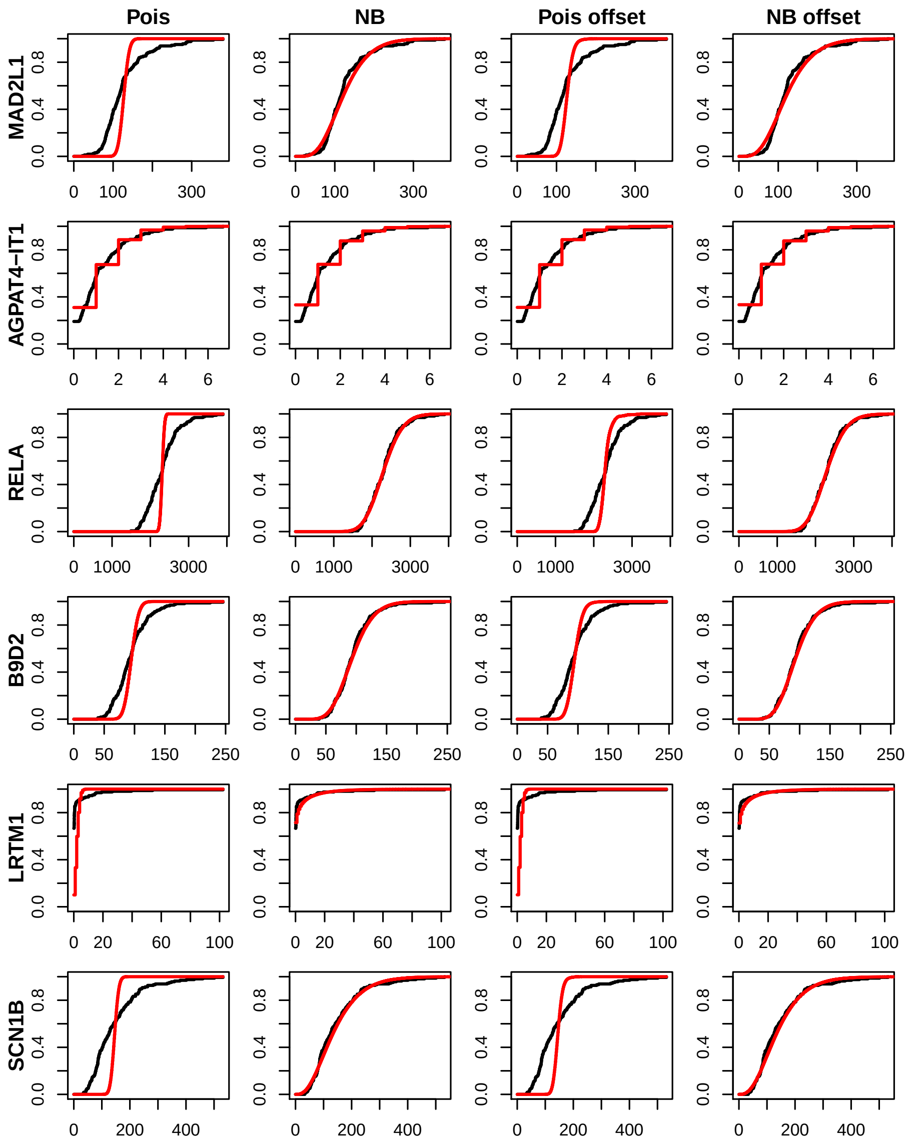

Figure E: **Cumulative distribution functions of RNA-Seq from the application TCGA for randomly selected genes.** Each row represents one gene, and each column represents one model. It is of interest how close the fitted distributions (red) come to the empirical distributions (black). Whether library sizes are ignored (columns 1 and 2) or an offset is included (columns 3 and 4), the negative binomial distribution with a free dispersion parameter has a much better fit than the Poisson distribution.

SDF4	VPS13D	KPNA6	TACSTD2	PRKAB2	NUCKS1
RER1	CTRC	ZBTB8A	ROR1	HIST2H2AB	FAM72A
NPHP4	FBXO42	TEKT2	SYDE2	PSMD4	RASSF5
ACOT7	SZRD1	THRAP3	EPHX4	BGLAP	HLX
TAS1R1	CNR2	ZMPSTE24	CCDC18	NUF2	MRPL55
ZBTB48	SRSF10	PPCS	GPR88	ADCY10	EXOC8
CAMTA1	TMEM50A	ERMAP	PSRC1	TNFSF4	OR2L13
PARK7	SEPN1	CFAP57	FAM19A3	RC3H1	
TARDBP	PIGV	TMEM125	TRIM33	ASTN1	
CLCN6	GPN2	SZT2	NRAS	DHX9	
KIAA2013	PPP1R8	KLF17	ATP1A1	PRG4	

Table E: **List of gene symbols.** In the application TCGA these genes are insignificant in both individual tests but significant in the joint test at a false discovery rate of 5%. Their expression is associated with methylations and copy numbers jointly, but with neither of them individually.

Bibliography

Goeman, J., van de Geer, S., de Kort, F., and van Houwelingen, H. (2004). "A global test for groups of genes: testing association with a clinical outcome", *Bioinformatics*, Vol. 20, pp. 93-99.

Lappalainen, T., Sammeth, M., Friedländer, M., 't Hoen P., Monlong J., Rivas M., et al. (2013). "Transcriptome and genome sequencing uncovers functional variation in humans", *Nature*, Vol. 501, pp. 506-511.

le Cessie, S., and van Houwelingen, H. (1995). "Testing the fit of a regression model via score tests in random effects models", *Biometrics*, Vol. 51, pp. 600-614.

Li, B., and Dewey, C. (2011). "RSEM: accurate transcript quantification from RNA-Seq data with or without a reference genome", *BMC Bioinformatics*, Vol. 12, pp. 323.

MD Anderson Cancer Center (2016). "TCGA Batch Effects Tool". Available from http://bioinformatics.mdanderson.org/tcgambatch/.

The Cancer Genome Atlas Research Network, Weinstein, J., Collisson, E., Mills, G., Shaw, K., Ozenberger, B., Ellrott, K., Shmulevich, I., Sander, C., and Stuart, J. (2013). "The Cancer Genome Atlas Pan-Cancer analysis project", *Nature Genetics*, Vol. 45, pp. 1113-1120.

Wang, K., Singh, D., Zeng, Z., Coleman, S., Huang, Y., Savich, G., He, X., Mieczkowski, P., Grimm, S., Perou, C., MacLeod, J., Chiang, D., Prins, J., and Liu, J. (2010). "MapSplice: Accurate mapping of RNA-seq reads for splice junction discovery", *Nucleic Acids Research*, Vol. 38, pp. e178.
